# Tuberous sclerosis complex–associated kidney disease in children

**DOI:** 10.1007/s00467-024-06642-9

**Published:** 2025-01-15

**Authors:** Matko Marlais, Djalila Mekahli

**Affiliations:** 1 Department of Paediatric Nephrology, Great Ormond Street Hospital for Children NHS Foundation Trust, Great Ormond Street, London, WC1N 3JH UK; 2 UCL Great Ormond Street Institute for Child Health, University College London, London, UK; 3 PKD Research Group, Laboratory of Ion Channel Research, Department of Cellular and Molecular Medicine, KU Leuven, Louvain, Belgium; 4 Department of Paediatric Nephrology, University Hospitals Leuven, Louvain, Belgium

**Keywords:** Tuberous sclerosis, Imaging, Kidney, Child, Genetics

## Abstract

Tuberous sclerosis complex (TSC) is an autosomal dominant disorder which can have manifestations in the kidneys, along with other organ systems. Children with TSC may develop kidney lesions at any point during childhood, and typically these are angiomyolipomata (AML) and/or kidney cysts. Children may also have hypertension associated with TSC-associated kidney disease, and rarely reduced kidney function. New guidelines for the management of TSC-associated kidney disease in children and adults were published in 2024. This educational review summarises the relevant clinical aspects of these guidelines for paediatric nephrologists through a series of four clinical cases. These cases cover management of hypertension, frequency of follow-up and frequency of kidney imaging. Difficult clinical scenarios are reviewed, such as the management of *TSC2-PKD1* contiguous gene syndrome and the management of large AMLs in children with TSC.

**Graphical abstract:**

**Figure:**
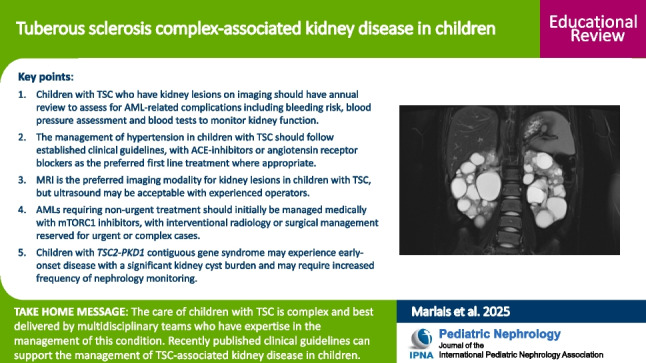
A higher resolution version of the Graphical abstract is available as Supplementary information

**Supplementary Information:**

The online version contains supplementary material available at 10.1007/s00467-024-06642-9.

## Introduction

Tuberous sclerosis complex (TSC) is an autosomal dominant disorder affecting multiple body systems. It is characterised by the growth of proliferative lesions in various organs, including the kidneys. The incidence of TSC is estimated at 1 in 5800 live births [1] and it can be a challenging disorder to diagnose as there is often no family history and the clinical phenotype can be variable [2].

Infants born with TSC rarely present with the kidney phenotype as the predominant problem of TSC; rather, issues such as epilepsy, developmental delay and cardiac rhabdomyomas predominate the infant and young child presentation [3]. The kidney phenotype in TSC includes angiomyolipomata (AML), kidney cysts and renal cell carcinoma (RCC), with the latter being uncommon during childhood. In adults, TSC-associated chronic kidney disease (CKD) is a significant cause of morbidity and mortality [1].

In 2024, clinical practice recommendations for the management of TSC-associated kidney disease in children and adults were published as a consensus statement from the ERKNet Working Group for Autosomal Dominant Structural Kidney Disorders and the ERA Genes & Kidney Working Group [4]. In this educational review, we explore these clinical practice recommendations with a focus on those most relevant to paediatric nephrology practice through a series of clinical scenarios.

## Case 1—hypertension management

A 12-year-old boy is referred to the paediatric nephrology clinic by his neurologist. He has TSC and has been under routine follow-up with the neurology team since infancy, presenting to medical care with seizures in the first year of life. His epilepsy is well controlled on levetiracetam and he has a mild learning disability. His neurologist refers him as his blood pressure (BP) is consistently > 95th centile for age, height and sex. TSC management is multidisciplinary, and therefore the initial specialist seeing a child must consider which other sub-specialists the child should be referred to in order to manage other organ manifestations.

Further exploration of his case during initial paediatric nephrology consultation reveals that he has a single pathogenic variant in *TSC2*; there is no family history. During initial work-up, he is found to be consistently hypertensive with office BP readings > 95th centile but otherwise clinically well. He has a kidney ultrasound (US) which finds multiple AMLs and cysts bilaterally with his largest AML currently 9 mm in diameter, with kidney size approximately 2 standard deviations above the mean. Initial blood tests show a normal serum creatinine of 48 umol/L.

In children with TSC, there is no evidence to suggest that hypertension should be managed any differently to current established guidelines such as the 2016 ESH guidelines [5] or the 2017 AAP guidelines [6]. This includes thresholds for starting treatment and BP thresholds to target when optimising treatment. Children with TSC and hypertension have CKD1 at least by virtue of their diagnosis, and therefore BP goals should be to target towards the 50th percentile (rather than targeting less than 90th percentile). It is important, however, to consider that BP measurement can be more challenging in children and young people with TSC. A significant proportion of individuals with TSC under paediatric care will have associated learning disability or autism spectrum disorder, and this can make accurate BP measurement in the outpatient clinic setting more difficult. Therefore, before commencing medication, it is prudent to review home BP measurement in a relaxed setting if possible, or perform 24-h ambulatory BP monitoring (ABPM).

Based on the above clinical scenario, current guidelines would support medical treatment of hypertension in this case, and the medication of choice in children with TSC would be an ACE inhibitor or angiotensin receptor blocker (ARB) unless there is any contraindication [4]. Although there is no specific evidence in children with TSC, the presence of cysts and the evidence of renin–angiotensin–aldosterone activation in cystic kidney diseases makes these a rational first-line agent.

The decision to initiate treatment may also be augmented through the use of 24-h ABPM which can be useful in certain settings, such as suspected white-coat hypertension. This, along with home BP monitoring, can also be useful in ongoing hypertension management to optimise treatment. We suggest that all children with TSC who have an office BP consistently > 95th centile should have a 24-h ABPM to confirm this. If there is evidence of hypertension, then screening for other target organ complications such as echocardiography or ophthalmology review should be considered.

## Case 2—imaging and follow-up frequency in children with TSC-associated kidney disease

A 7-year-old girl has a clinical diagnosis of TSC based on the presence of multiple cortical tubers and hypomelanotic macules. Despite extensive genetic testing in a laboratory with expertise in TSC genetics, no mutation was identified and she is diagnosed clinically as having TSC based on the criteria in Table 1, which are adapted from international guidelines [7]. There are a wide variety of variants in *TSC1* or *TSC2* which can cause the disease, but assessment of pathogenicity of these variants is not straightforward in all cases. A pathogenic variant cannot be identified in 10–15% of individuals with a clinical diagnosis of TSC, and in many such cases there is mosaicism for a pathogenic variant (usually *TSC2*). For this reason, expert genetic interpretation and genetic counselling, including screening other family members, is advised.

**Table 1:** Diagnostic criteria for tuberous sclerosis complex, adapted from [7]

Major criteria	Minor criteria
Hypomelanotic macules (≥ 3; at least 5 mm diameter)	“Confetti” skin lesions
Angiofibroma (≥ 3) or fibrous cephalic plaque	Dental enamel pits (≥ 3)
Ungual fibromas (≥ 2)	Intraoral fibromas (≥ 2)
Shagreen patch	Retinal achromic patch
Multiple retinal hamartomas	Multiple renal cysts
Multiple cortical tubers and/or radial migration lines	Non-renal hamartomas
Subependymal nodule (≥ 2)	Sclerotic bone lesions
Subependymal giant cell astrocytoma	
Cardiac rhabdomyoma	
Lymphangiomyomatosis^∗^	
Angiomyolipomas (≥ 2)^∗^	

Definite TSC: 2 major features or 1 major feature with 2 minor features.

Possible TSC: either 1 major feature or ≥ 2 minor features

This girl has been referred to the paediatric nephrology clinic for monitoring. Her neurologist has already undertaken a kidney US as per current guidelines [4] but this has not identified any kidney lesions and is reported as normal. In such a case, the guidelines no longer advise annual blood tests to monitor kidney function. If there is no evidence of TSC-associated kidney disease on imaging, it is reasonable to delay blood tests to check kidney function until adolescence or adulthood, but annual BP measurement should still be performed. This is because the serum creatinine and therefore estimated GFR in children with TSC is often normal, with CKD stages 2–3 uncommon until adulthood [8, 9].

This girl continues under paediatric nephrology follow-up, as the clinical guidelines advise repeat kidney imaging every 1–3 years to monitor appearances, either with MRI or US in the hands of an expert operator. When she is seen aged 13 years, she is found to have some small (< 1 cm diameter) AMLs on her kidney ultrasound scan; her kidneys are normal in size overall, with 9.5 cm left and 9.7 cm right kidney, which is in the normal range of kidney size centiles. This is quite a typical situation, as the appearance of kidney lesions in TSC is known to accelerate during adolescence.

In this situation, the appearance of kidney lesions on imaging requires the clinician to review their follow-up frequency. While this child may have been seen every 2–3 years, if there are kidney lesions seen, the follow-up frequency should be changed to annual. During the annual nephrology review for children with TSC-associated kidney lesions, we recommend assessment for AML-related complications including bleeding risk, BP assessment, and blood tests to monitor kidney function [4]. In some children, especially those with low muscle mass (e.g. those with severe neurological manifestations), cystatin C–based formulae can give a better estimate of eGFR.

The optimal interval between imaging may also be adjusted, as while there are no kidney lesions, it may be reasonable to delay next imaging by 2–3 years in children; this may need to be reviewed when kidney lesions arise. The rationale for this increased imaging frequency is primarily to assess lesion growth, which may be indicative of AML bleeding risk [10].

**Fig. 1:**
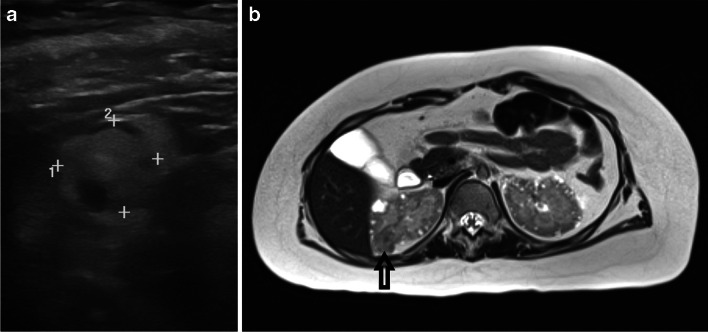
**a**, **b** Comparison of kidney lesion visualisation in TSC between US and MRI. Arrow on T2-weighted MRI image in right panel shows the lesion which is represented on left US panel, lesion measures 18 mm × 17 mm in Fig. 1a

Magnetic resonance imaging (MRI) is the imaging modality of choice for children with TSC. CT is avoided where possible in order to limit exposure to ionising radiation and to limit exposure to intravenous contrast which may be nephrotoxic. However, CT may be required in urgent situations or in patients who are unable to have MRI due to implanted devices. US in the hands of an expert operator is an acceptable alternative to MRI. US has the benefit of being cheaper and more readily available than MRI but operator-dependent and can be challenging in children with TSC who may suffer from autism spectrum disorder or other behavioural difficulties. Based on its superior ability to differentiate lipid-rich and lipid-poor masses, MRI is the preferred modality for detection and monitoring lesions in children with TSC. The clinical guidelines recognise that for children an US performed by an expert operator experienced in lesion detection in TSC may be an acceptable alternative, especially in younger children [4]. However, US can miss fat-poor AMLs and therefore we advise MRI is performed at least once during childhood, even if other imaging monitoring is undertaken with US. Figure 1a and b show a comparison of lesion visualisation on US and MRI in the same patient, where body habitus means that lesion visualisation, measurement and characterisation can be significantly aided with MRI. This case demonstrates the obesity which is a feature in some children with TSC and can make US visualisation of lesions even more challenging.

In children with TSC who may be undergoing annual MRI brain for monitoring with their neurologist, we would advise efforts are made to combine this with an annual MRI abdomen to monitor the kidneys. This is especially important if the child requires a general anaesthetic for MRI, to avoid multiple anaesthetics. For this reason, management of TSC in children should be multidisciplinary in expert centres to allow for streamlined and patient-centred care [11].

## Case 3—*TSC2–PKD1* contiguous gene syndrome

**Fig. 2:**
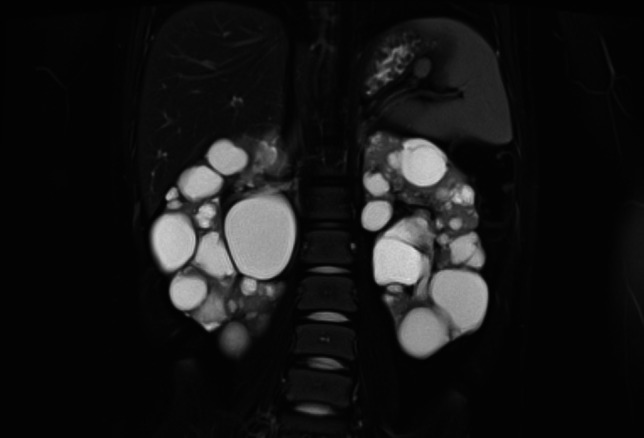
MRI images showing multiple bilateral renal cysts in an 8-year-old girl with *TSC2–PKD1* contiguous gene syndrome (MRI acquisition sequence: T2-weighted with fat saturation)

A 2-year-old girl is referred by her cardiologist who has been following her up after the presence of cardiac rhabdomyoma from birth. She is found to have innumerable cysts in both kidneys on ultrasound and her kidneys are both grossly enlarged with right kidney 11.9 cm in length and left kidney 13.7 cm in length (95th centile for kidney length by age is 8.2 cm), with largest cysts 31 mm diameter and 34 mm diameter, respectively. Genetic testing reveals that she has a genomic deletion in *TSC2* which involves *PKD1*. Figure 2 shows the MRI images for this girl when she is 8 years old.

Both the *PKD1* and *TSC2* genes are located adjacent to each other on chromosome 16. This means that larger deletions of either gene can affect the other, leading to a more severe kidney phenotype which typically presents as early and potentially severe cystic kidney disease [12, 13]. The kidney phenotype in *TSC2–PKD1* contiguous gene syndrome can start as early as the neonatal period [14].

Although evidence in children with *TSC2–PKD1* contiguous gene syndrome is limited, the knowledge we have suggests that monitoring and follow-up frequency may need to be adapted in such cases. We advise a more regular approach to BP monitoring with an increased frequency, as hypertension may occur earlier in those cases where there is a high cyst burden. Blood pressure treatment targets should also be lower in children with *TSC2–PKD1* contiguous gene deletion who are on medical management for hypertension, as this may have a renoprotective effect and help to preserve GFR based on studies of other cystic kidney diseases such as autosomal dominant polycystic kidney disease (ADPKD) [15, 16].

Kidney function monitoring and blood tests may also need to be performed more frequently in children with *TSC2–PKD1* contiguous gene syndrome and there is a risk of kidney failure. There is interest in whether mechanistic target of rapamycin complex 1 (mTORC1) inhibitors, which are used for the management of large AMLs in TSC, may be beneficial in patients with TSC who have a significant cyst burden. The current clinical guidelines do not make a recommendation on this as there is insufficient evidence, but there are small series showing a reduction in overall cyst burden with mTORC1 inhibition [17]. There are also no current data on the use of tolvaptan in patients with TSC who have a significant cyst burden, despite the evidence of benefit in patients with ADPKD.

## Case 4—large angiomyolipomata

A 13-year-old boy initially presented during infancy with infantile spasms and was diagnosed with TSC and has a *TSC2* mutation. He is under regular follow-up and is found to have an AML which is 33 mm in diameter on an annual ultrasound scan. The lesion appears typical for an AML on ultrasound, with hyperechogenicity and a homogeneous appearance. MRI was not performed in this case as the lesion appeared typical, but MRI can be helpful for lesions of this size and can differentiate fat-poor lesions. The lesion has grown 4 mm on ultrasound since the previous year.

While the overall risk of spontaneous bleeding from AMLs in TSC is relatively low, estimated at around 5% [18], there are certain characteristics which increase the risk of spontaneous bleeding. These include *TSC2* mutation, female sex, age > 15 years, total diameter > 30 mm and presence of intralesional aneurysms > 5 mm [4]. Therefore, once a lesion is > 30 mm in diameter, consideration can be given as to whether this lesion may benefit from treatment.

Various treatment options exist for large AMLs, including medical, interventional and surgical. While historically surgical procedures were undertaken more commonly in TSC, previous nephrectomy is associated with an increased risk of CKD and kidney failure in adults with TSC [19, 20]. Since the availability of medical therapy with mTORC1 inhibitors, there is an opportunity to reduce this risk of CKD and kidney failure, which is especially important for children who have more years ahead of them. The guidelines therefore recommend that for AMLs requiring non-urgent treatment, medical treatment with an mTORC1 inhibitor should be the first-line management option [4].

Medical therapy for large AMLs is centred around trying to reduce the risk of complications, especially the risk of spontaneous bleeding. The EXIST-1 and EXIST 2 studies have shown the benefit of mTORC1 inhibition in patients who have AMLs > 30 mm in diameter [21, 22]. Based on this, and in order to reduce the long-term risk of CKD and kidney failure, the clinical guidelines suggest that in children with TSC who have a growing AML > 30 mm, mTORC1 inhibitor treatment should be considered [4].

If mTORC1 inhibition is started, the recommended starting dose is 2.5 mg/m^2^ in children. mTORC1 inhibition is generally well tolerated with limited side effects, but it does require monitoring of blood tests for renal function and electrolytes, liver function and glucose, along with urine monitoring for proteinuria [4]. Once treatment is started, the guidelines recommend waiting 12 months to assess for response in typical AMLs. The median time for AML response in the EXIST-2 study was 3 months [22]; therefore, there is little value in repeating imaging to check for response at an interval of less than 6 months. The response to treatment with mTORC1 inhibitors can be defined as growth arrest of the AML or reduction in the speed of growth of the AML. If there is a clear response to mTORC1 inhibitor treatment and the patient is tolerating the treatment, the guidelines recommend continuing with mTORC1 inhibitor therapy. If there is no obvious response to treatment within 12 months, this must be reviewed, adherence checked and consideration given as to whether the lesion treated is in fact a typical AML [4]. A lack of response of a suspected AML to mTORC1 inhibitor treatment may be due to several factors, but may also raise the possibility of kidney cancer in a growing lesion [23].

**Fig. 3:**
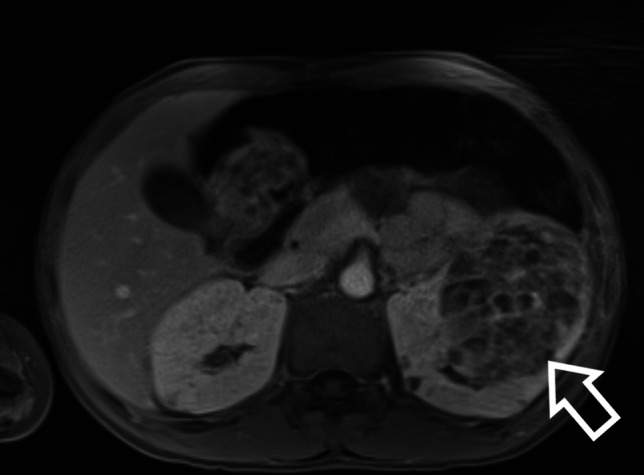
MRI of a large fat-poor AML, eventually confirmed histologically after biopsy was undertaken for rapid growth (MRI acquisition sequence: T1-weighted with fat saturation)

Although AMLs typically have intralesional fat which is seen on imaging, the amount of fat within them can vary. Fat-poor lesions may represent up to 1/3 of AMLs [24, 25]. RCC is extremely rare in children with TSC, although case reports do exist. Figure 3 shows an example of a large fat-poor AML on MRI imaging (from a different patient to the original case), eventually confirmed as an AML histologically after a biopsy was undertaken for rapid growth of the lesion.

The guidelines do not recommend routine kidney lesion biopsy for all fat-poor lesions on imaging; this is especially important for children where RCC is extremely rare. mTORC1 inhibition is recommended as first-line treatment for fat-poor lesions that do not require urgent intervention, and kidney lesion biopsy reserved for those cases with rapid (> 5 mm/year) growth or those lesions which do not respond to mTORC1 inhibitor therapy [4]. While mTORC1 inhibitor therapy is available in many countries, there are some countries where it is not reimbursed for kidney indications and only reimbursed in cases of subependymal giant cell astrocytoma (SEGA) due to a lack of studies, and this creates challenges with equity of access for patients.

## Conclusions

Children with TSC can have several kidney manifestations, including hypertension and kidney lesions on imaging. Some children will have a more severe kidney phenotype with earlier onset, especially those with *TSC2–PKD1* contiguous gene syndrome. Historically, the management and follow-up of children with TSC was variable from a nephrological perspective, and many children with TSC still do not receive appropriate monitoring from a kidney perspective.

New clinical practice guidelines published in 2024 highlight important management aspects of TSC in children. These guidelines review follow-up and monitoring frequency for nephrologists and management of hypertension, as well as reviewing imaging frequency and modalities. RAAS inhibition is the first line for management of hypertension. mTORC1 inhibitors are now the preferred management option for children with TSC and AMLs that require non-urgent treatment. The care of children with TSC is complex and best delivered by multidisciplinary teams who have expertise in the management of this condition.

## Key summary points

1. Children with TSC who have kidney lesions on imaging should have annual review to assess for AML-related complications including bleeding risk, blood pressure assessment and blood tests to monitor kidney function.
2. The management of hypertension in children with TSC should follow established clinical guidelines, with ACE inhibitors or angiotensin receptor blockers as the preferred first-line treatment where appropriate.
3. MRI is the preferred imaging modality for kidney lesions in children with TSC, but ultrasound may be acceptable with experienced operators.
4. AMLs requiring non-urgent treatment should initially be managed medically with mTORC1 inhibitors, with interventional radiology or surgical management reserved for urgent or complex cases.
5. Children with *TSC2–PKD1* contiguous gene syndrome may experience early-onset disease with a significant kidney cyst burden and may require increased frequency of nephrology monitoring.

## Multiple choice questions

Answers are given following the reference list.

1. For an AML in a child with TSC, which of these features is NOT a bleeding risk factor?
  - Size > 30 mm
  - Female sex
  - Fat-poor appearance
  - Age > 15 years
  - *TSC2* mutation
2. In a child with TSC who has some kidney lesions on ultrasound, what is the standard nephrological monitoring interval?
  - 3-monthly
  - 6-monthly
  - 12-monthly
  - Every 2 years
3. What is the first-line class of medications to treat hypertension in children with TSC?
  - Calcium channel blocker
  - ACE inhibitor
  - Beta-blocker
  - Diuretic
  - Alpha-blocker
4. What is the median response time to mTORC1 inhibitor therapy for kidney AMLs in TSC?
  - 3 months
  - 4 months
  - 5 months
  - 6 months

## Supplementary Information

Below is the link to the electronic supplementary material.

Graphical abstract (PPTX 402 KB)
