# Supplementary material for: Tuberous sclerosis complex–associated kidney disease in children
Source: Pediatr Nephrol. 2025 Jan 15;40(6):1871–7. doi: 10.1007/s00467-024-06642-9 (PMC12031771; doi:10.1007/s00467-024-06642-9)
Supplement: Supplementary file 1 — Graphical abstract (PPTX 402 KB) [file 467_2024_6642_MOESM1_ESM.pptx]

## Slide 1
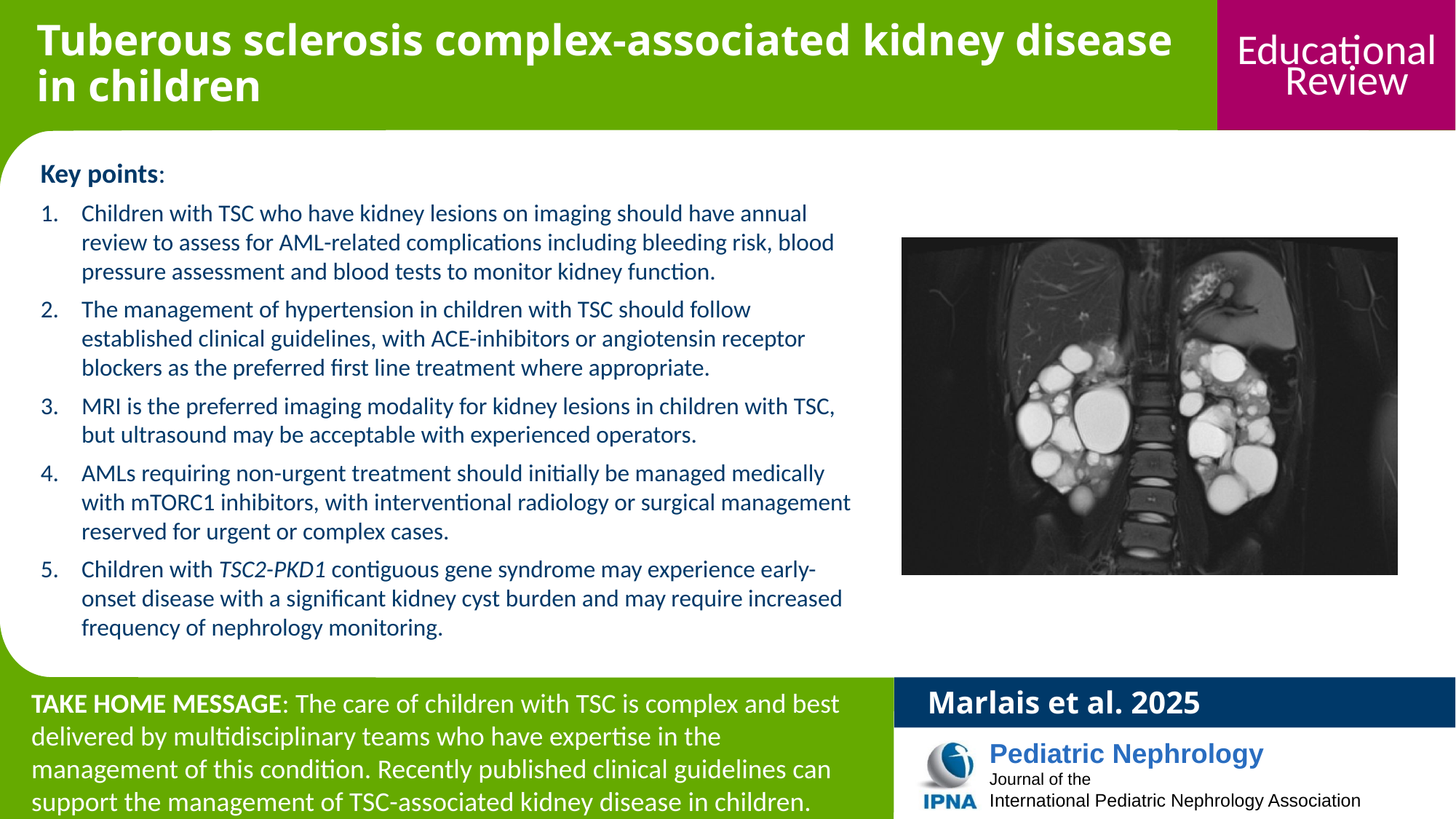

Tuberous sclerosis complex-associated kidney disease in children
Key points:
Children with TSC who have kidney lesions on imaging should have annual review to assess for AML-related complications including bleeding risk, blood pressure assessment and blood tests to monitor kidney function.
The management of hypertension in children with TSC should follow established clinical guidelines, with ACE-inhibitors or angiotensin receptor blockers as the preferred first line treatment where appropriate.
MRI is the preferred imaging modality for kidney lesions in children with TSC, but ultrasound may be acceptable with experienced operators.
AMLs requiring non-urgent treatment should initially be managed medically with mTORC1 inhibitors, with interventional radiology or surgical management reserved for urgent or complex cases.
Children with TSC2-PKD1 contiguous gene syndrome may experience early-onset disease with a significant kidney cyst burden and may require increased frequency of nephrology monitoring.
Marlais et al. 2025
TAKE HOME MESSAGE: The care of children with TSC is complex and best delivered by multidisciplinary teams who have expertise in the management of this condition. Recently published clinical guidelines can support the management of TSC-associated kidney disease in children.
